# Ectopic molar pregnancy: a case report

**Published:** 2012-04-04

**Authors:** Najoua Bousfiha, Sanaa Erarhay, Adnane Louba, Hanan Saadi, Chahrazad Bouchikhi, Abdelaziz Banani, Hind El Fatemi, Med Sekkal, Afaf Laamarti

**Affiliations:** 1 Department of gynecology obstetric I, Teaching Hospital, Hassan II, Fez, Morocco; 2 Department of anatomopathology, Teaching hospital Hassan II, Fez, Morocco

**Keywords:** Ectopic molar pregnancy, molar pregnancy, Ectopic pregnancy, Morocco

## Abstract

The incidence of hydatidiform moles is 1 per 1,000 pregnancies. Ectopic pregnancy occurs in 20 per 1,000 pregnancies. Thus, the incidence of the ectopic molar gestation is very rare. We report a case of tubal molar pregnancy diagnosed at the systematic histology exam of an ectopic pregnancy. We report the case of 32 years old nulliparus women who presented a vaginal bleeding, lower abdominal pain and 6 weeks amenorrhea corresponding to the last menstrual period. At the clinical examination, the arterial pressure was 100/60 mmHG. The gynecological examination was difficult because of lower abdominal pain. Serum gonadotropin activity was 3454 ui/l. Pelvic ultrasound revealed an irregular echogenic mass in the left adnexa. Diagnostic laparoscopy revealed a left-sided unruptured ampullary ectopic pregnancy. A left laparoscopic salpingectomy was performed. The systematic histologic test identified an ectopic partial molar pregnancy, which was confirmed by DNA ploidy image analysis. The patient was followed with weekly quantitative B-hCG titers until three successive B-hCG levels were negative. It is pertinent that clinicians take routine histological examination of tubal specimens in ectopic pregnancy very seriously in order to diagnose cases of ectopic molar gestations early and mount appropriate post treatment surveillance.

## Introduction

The incidence of hydatidiform moles is 1 per 1,000 pregnancies [1]. Ectopic pregnancy occurs in 20 per 1,000 pregnancies [2]. Thus, the ectopic molar gestation is very rare.

## Patient and case report

Madame SB, 32 years old, Moroccan women, married, nulliparous, without notable medical history, presented to the gynecological emergency unit of the teaching hospital Hassan II, for vaginal bleeding, lower abdominal pain and 6 weeks amenorrhea corresponding to her last menstrual period. No vagal symptoms were reported. At the clinical examination, the arterial pressure was 100/ 60 mmHg.The external genitalia and cervix were normal. The uterus was normal in size and position. The examination of adnexa was difficult because of lower abdominal pain.

**Figure 1:**
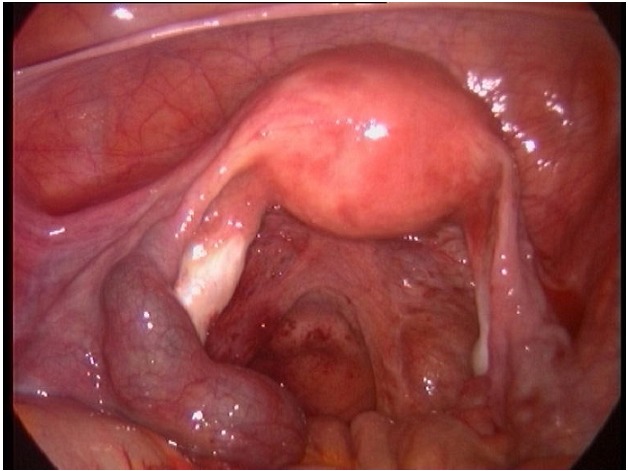
The left-sided unruptured ampullary ectopic pregnancy at laparoscopy

Serum gonadotropin activity was 3454 ui/l. Pelvic ultrasound revealed an irregular echogenic mass in the left adnexa (1.5cm × 2 cm). Diagnostic laparoscopy revealed a left-sided un-ruptured ampullary ectopic pregnancy (1.5 cm × 2 cm). The uterus, left ovary, right tube and ovary were normal (Figure 1).

**Figure 2:**
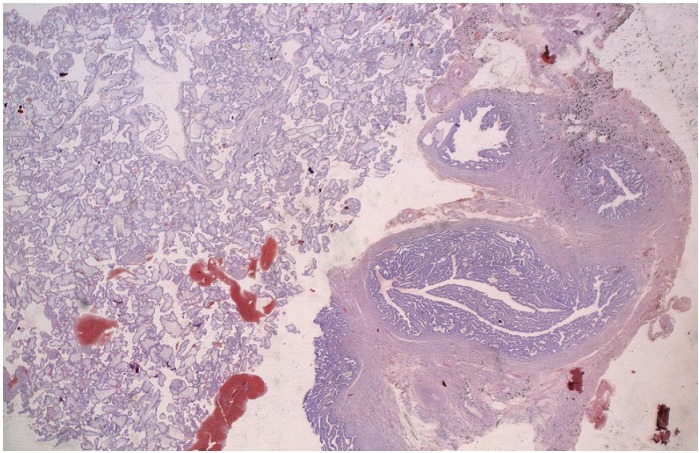
Developing of enlarged villi in the lumina of fallopian tube (HES x 10)

**Figure 3:**
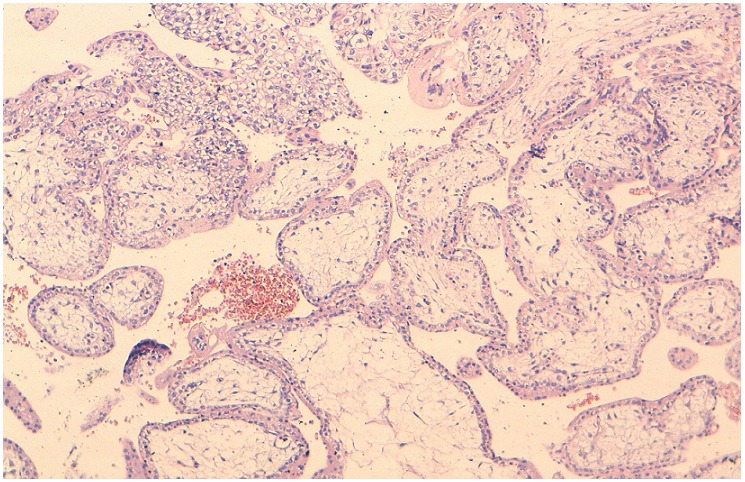
Enlarged villi with stromal edema and proliferation of cytotrophoblast and syncytiotrophoblast (HES x 100)

A left laparoscopic salpingectomy was performed. There was negligible loss of blood. The postoperative course was uneventful. The systematic histologic test identified ectopic partial molar pregnancy, which was confirmed by DNA ploidy image analysis (Figure 2, Figure 3). The patient was followed with weekly quantitative β-hCG titers until three successive β-hCG levels were negative. She was advised to avoid pregnancy for 6 months and was started on oral contraceptive pills.

## Discussion

Hydatidiform mole is basically an abnormal conceptus, due to abnormal fertilization which can be sub-classified into complete and partial moles based on morphological, pathological, and genetic differences [1, 2].

In a complete mole, the chromosomal complement is 46,XX with the genome paternal in origin. This is usually caused by fertilization of an empty ovum by a haploid spermatozoon, which subsequently duplicates. Occasionally cases occur by fertilization with two sperm [3]. In contrast, partial moles arise from dispermic fertilization of a haploid ovum, resulting in a triploid genome.

Histologically, molar pregnancy is an abnormal gestation characterized by the presence of hydropic change affecting some or all of the placental villi, accompanied by circumferential proliferation of trophoblasts. Nonmolar hydropic abortions are common; it is clinically important to distinguish molar pregnancies from nonmolar hydropic changes, because the former has the potential of causing persistent trophoblastic disease [4]. Furthermore, the blighted ovum is a common feature in ectopic pregnancy and can easily be misinterpreted as a true hydatidiform mole [5]. However, the early swellings of the placental villi do not constitute a true hydatidiform mole. The salient diagnostic criteria of nonmolar pregnancy are liquefaction and edema of villous stroma, scantiness or absence of the villous blood vessels, and trophoblastic proliferation [5].

Genotyping and chromosome in situ hybridization can provide reliable adjunct to histology for the classification of a hydatidiform mole, especially in cases with difficult histological evaluation [6]. Molecular techniques are only of value in distinguishing a diploid from a triploid mole once the diagnosis is made histologically [6, 7]; it does not help to distinguish complete mole from hydropic abortion. Although -hCG levels are elevated in tubal molar pregnancies, they are generally in the lower range, because implantation in the fallopian tube might preclude adequate vascularization, thereby leading to low levels of hCG. There is no distinctive difference in -hCG levels between molar tubal pregnancies and ectopic pregnancy. Thus, an early ectopic molar pregnancy is not distinguishable from a nontrophoblastic tubal pregnancy on the basis of hCG levels [8].

Gestational choriocarcinoma associated with ectopic pregnancy is extremely rare event: its theoretic incidence is one in 5033 tubal pregnancies. The prognosis of choriocarcinoma is better in the tube than in the uterus because molar pregnancy in the tube is removed and not left intact, as in the uterus [9].

## Conclusion

It is pertinent that clinicians take routine histological examination of tubal specimens in ectopic pregnancy very seriously in order to diagnose cases of ectopic molar gestations early and mount appropriate post treatment surveillance.
